# Temperature-Dependence of Predator-Prey Dynamics in Interactions Between the Predatory Fungus *Lecophagus* sp. and Its Prey *L. inermis* Rotifers

**DOI:** 10.1007/s00248-017-1060-5

**Published:** 2017-09-30

**Authors:** Edyta Fiałkowska, Agnieszka Pajdak-Stós

**Affiliations:** Institute of Environmental Sciences, Jagiellonian University, Gronostajowa 7, 30-387 Kraków, Poland

**Keywords:** Hyphomycetes, Conidia, Top-down control, Activated sludge, Wastewater treatment

## Abstract

Temperature is considered an important factor that influences the bottom-up and top-down control in water habitats. We examined the influence of temperature on specific predatory-prey dynamics in the following two-level trophic system: the predatory fungus *Lecophagus* sp. and its prey *Lecane inermis* rotifers, both of which originated from activated sludge obtained from a wastewater treatment plant (WWTP). The experiments investigating the ability of conidia to trap rotifers and the growth of fungal mycelium were performed in a temperature range that is similar to that in WWTPs in temperate climate. At 20 °C, 80% of the conidia trapped the prey during the first 24 h, whereas at 8 °C, no conidium was successful. The mycelium growth rate was the highest at 20 °C (*r* = 1.44) during the first 48 h but decreased during the following 24 h (*r* = 0.98), suggesting the quickest use of resources. At a medium temperature of 15 °C, the tendency was opposite, and the *r* value was lower during the first 48 h. At 8 °C, the growth rate was very low and remained at the same level even though numerous active rotifers were potentially available for the fungus. The temperature also influences the production of new conidia; on the 7th day, new conidia were observed in 96% of the wells at 20 °C, but no new conidia were observed at 8°C. These results show that the prey (rotifers)-predator (*Lecophagus*) dynamics in WWTPs is temperature-dependent, and a temperature of 8 °C is a strongly limiting factor for the fungus. Moderate temperatures ensure the most stable coexistence of the fungus and its prey, whereas the highest temperature can promote the prevalence of the predator.

**Electronic supplementary material:**

The online version of this article (10.1007/s00248-017-1060-5) contains supplementary material, which is available to authorized users.

## Introduction

Predacious fungi are an ecological group comprising different phylla, such as Ascomycota, Zygomycota, Basidiomycota, and Zoopagales [1]. Thus far, approximately 200 species of predatory fungi have been described in the literature. Most predatory fungi are soil organisms that prey on nematodes as either endoparasites or nematode-trapping fungi [1–3]. Some fungi are also able to feed on tardigrades, amoebae, and rotifers. Approximately, 40 predacious fungi/fungi-like organisms occur in water habitats. Scarce reports mentioned the presence of predacious fungi in activated sludge [4–8]. While much is known regarding the biology and systematics of fungi feeding on nematodes [1–3, 9], knowledge of biology and systematics of fungi preying on rotifers is rather limited, particularly regarding fungi that inhabit activated sludge [8].

Predacious fungi that feed on rotifers belong to the following two genera: *Zoophagus* and *Lecophagus*. The fungi that belong to the genus *Zoophagus* have been reported to feed mainly on loricated rotifers, whereas those belonging to the genus *Lecophagus* trap mainly bdelloid rotifers and tardigrades. Thus far, the only exception recently described is *Lecophagus vermicola*, which feed exclusively on nematodes [10]. All previously described species were isolated from wet habitats, such as moss, decaying plants on the banks of rivers and lakes, cyanobacterial mats, and animal feces [11–13], except for *L. vermicola*, which were isolated from bark fissures of *Platanus* and other trees [10]. In our recently published manuscript [8], we described the results of experiments conducted on a *Zoophagus* sp. isolated from activated sludge. The study showed that fungi can significantly reduce the number of *Lecane* and Bdelloidea individuals; however, *Lecane inermis* was the most affected [8]. The relationship between predacious fungi and rotifers in wastewater treatment plants (WWTPs) is of utmost importance because rotifers play a significant role in activated sludge. The loricated *Lecane* rotifers can reduce the growth of different filamentous bacteria species that are responsible for the highly disadvantageous phenomenon of activated sludge bulking and foaming [14–17], whereas bdelloid rotifers enhance floc formation and contribute to the reduction of excess sludge production [18, 19].

Temperature has been shown to greatly influence the growth and other life-history parameters of rotifers [20–23]. More detailed studies regarding the biology of *Lecane* rotifers originating from activated sludge have shown that these rotifers strongly depend on temperature. Studies investigating different clones of *L. inermis* have shown that generally, despite certain interclonal differences, their growth rate drastically decreases as the temperature decreases. A temperature of 8 °C, at which the value of the growth rate coefficient is nearly 0, appears to be critical for this species. Nevertheless, certain clones of *L. inermis* have a positive growth rate, even at such a low temperature [24]. Other experiments aiming to select rotifers that have better adapted to low temperatures and are capable of limiting the growth of filamentous bacteria have led to the selection of *L. tenuiseta* clones, which can proliferate at temperatures as low as 8 °C [25]. The abovementioned experiments were performed at a temperature range similar to that in WWTPs operating in temperate climate zones. As both species of *Lecane* are potential bulking and foaming control agents, knowledge regarding the conditions that contribute to their survival in activated sludge is necessary for optimizing their use as biological tools in real scale WWTPs.

Because it has been previously shown that a population of rotifers in activated sludge could be limited by predatory fungi and low temperatures, we designed a set of experiments to determine whether the growth of a predatory fungus isolated from activated sludge that belongs to the genus *Lecophagus* sp. depends on temperature. Furthermore, we aimed to characterize how temperature influences the susceptibility of rotifers *Lecane* sp. to the fungus.

## Materials and Methods

**Fig. 1:**
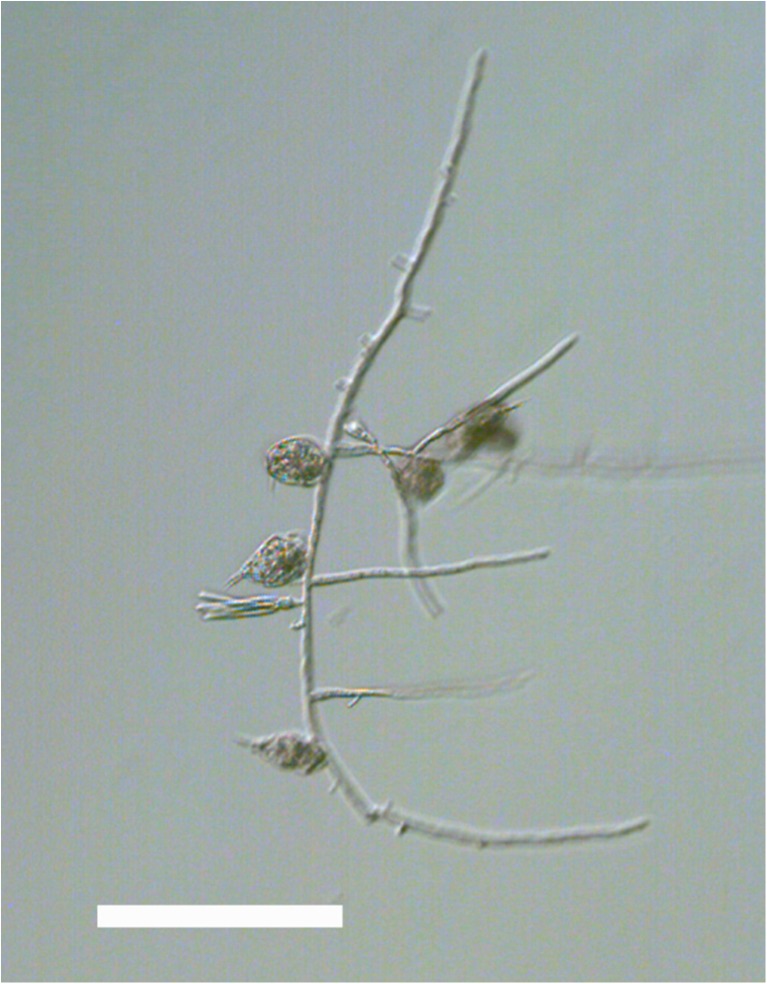
Exemplary piece of *Lecophagus* sp. mycelium with trapped rotifers and conidia growing on a conidiophore cell. Scale bar indicates 200 μm

**Fig. 2:**
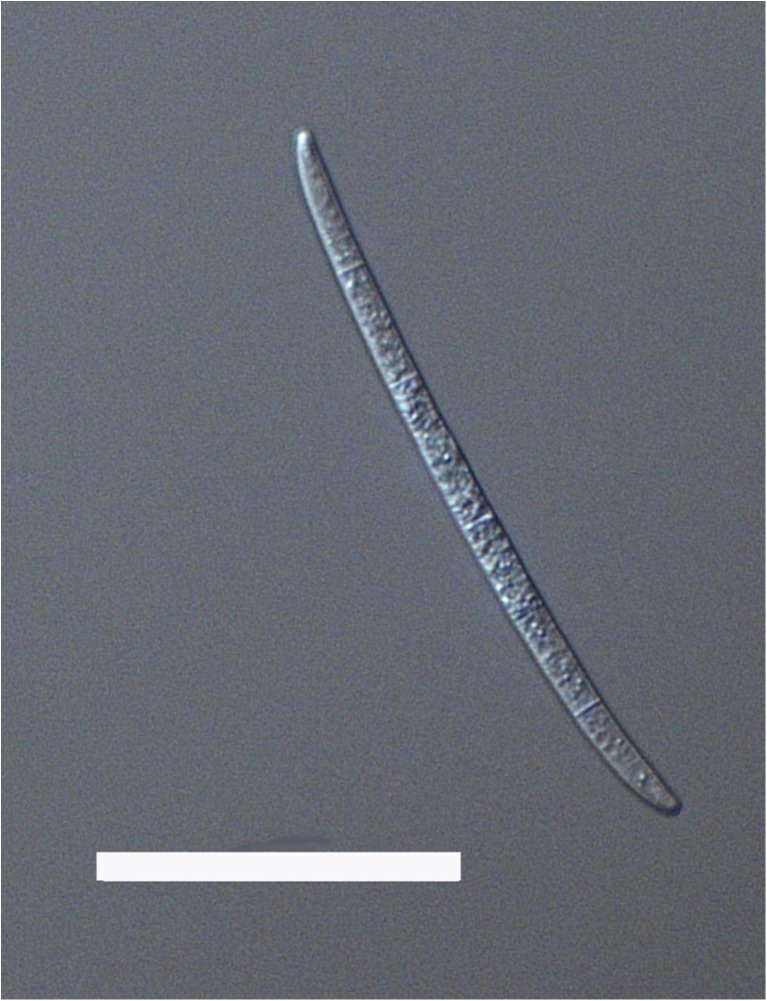
Conidium with visible septa. Scale bar indicates 50 μm

**Fig. 3:**
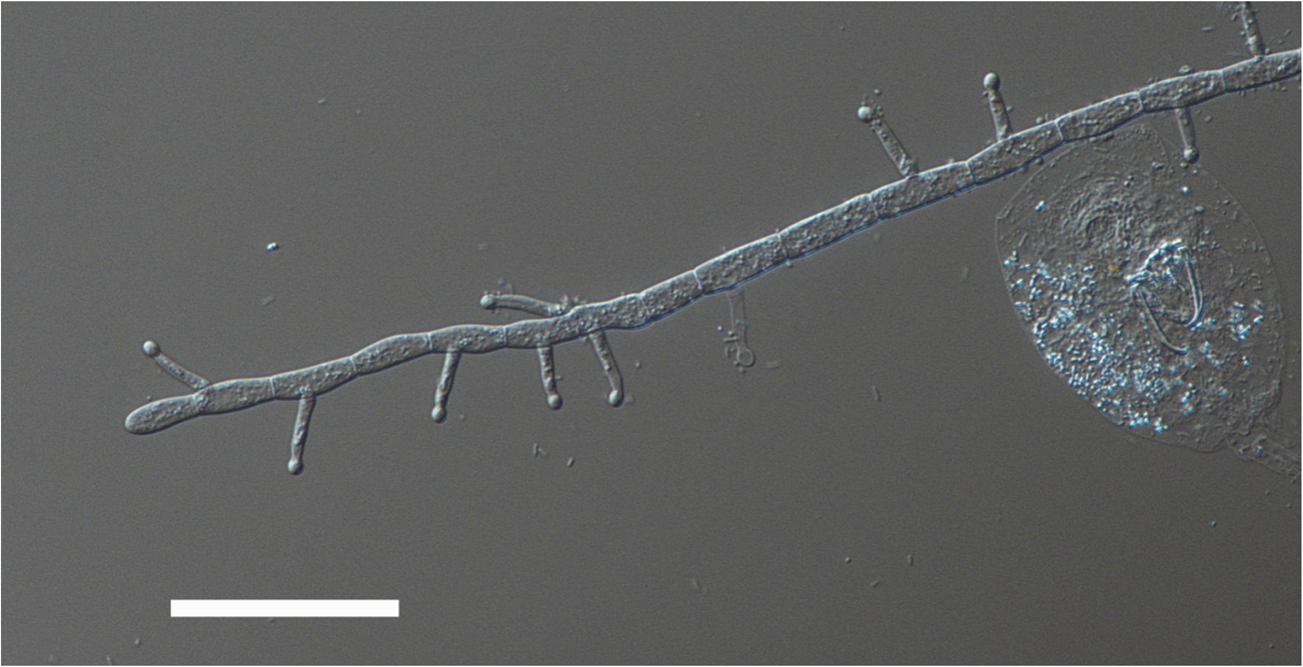
Fragment of the fungus mycelium with clearly visible septa and adhesive pegs. Scale bar indicates 50 μm

In this experiment, we used clonal populations of the predacious fungus *Lecophagus* sp. and rotifers *Lecane* sp. that were isolated from wastewater treatment plants in Southern Poland. The fungus was detected in a sample obtained from a small WWTP treating domestic waste. Some pieces of mycelium were then transferred to a Petri dish filled with Żywiec brand spring water, and the rotifers *L. inermis* were added as a food source. The dish was maintained in darkness at 20 °C. When the fungus produced conidia, some of them were transferred individually to separate wells in 12-well tissue culture plates and maintained similarly. One of the obtained clones, coded as Z1, was used in the experiment. We classified this fungus as *Lecophagus* according to a key provided by Dick [26], in which the main criterion distinguishing *Lecophagus* from *Zoophagus* is the septation of the mycelium. The fungus mycelium is approximately 6.5 μm wide, branched and septate (Fig. 1). The length of the segments is 15–21 μm. Conidiogenous cells lateral, bearing 2–5 conidia (Supplementary Material). The conidia septate, usually with 4–6 septa (Fig. 2). Most conidia are 4.9–6.6 μm wide and 95–125 μm long, but solitary conidia of 82.0 or 140 μm were also recorded. The adhesive pegs are broader at the base and clearly indented immediately below the rounded adhesive apex (Fig. 3). The pegs are 14–17 μm long. Because the biometric features do not entirely match any of the already described *Lecophagus* species, we decided to only use the genus name.

The rotifers *L. inermis* (clone Lt2.B5) were used as prey organisms. The clone Lt2.B5 was obtained from single individuals isolated from activated sludge samples. The samples were evenly distributed into the wells of tissue culture plates and maintained in darkness at 8 °C. Throughout 2 years, the rotifers exhibiting the highest growth rate were transferred to new wells. Then, single individuals were transferred to new wells to obtain the clonal populations. The clone adapted to lower temperature obtained using a previously described selective procedure was then cultured at 8 °C in Żywiec brand spring water and fed NOVO (nutrition powder used for rotifer mass culture, patent EP2993978(A1)) [27]. Using clones adapted to lower temperatures ensured the rotifer survival at each temperature used in the experiments.

## The Experimental Setup

Single conidia, which were approximately 100 μm long, and 10 μl of the medium were transferred to separate wells in three 24-well tissue plates using an automatic pipette. In total, 50 μl of the rotifer culture were inoculated into each well; thus, approximately 100 active individuals were present in each well. Then, 1 ml of Żywiec brand spring water was added. The plates were maintained in darkness at 8, 15, and 20 °C each.

After 24 h, we determined the number of conidia that managed to catch the rotifers, and the percentage of successful conidia was calculated at each temperature. After the following 24 h, the conidia found on the bottom were measured along with the mycelium that grew from the conidia. In certain cases, it was impossible to detect a conidium most likely because the conidia moved to the very edge of the well where they remained undetectable using an inverted microscope. The measurements were repeated after the following 24 h. After the fourth day of the experiment (96 h after the start of the experiment), the pieces of mycelium, particularly those in the wells maintained at 20 °C, were already too long and started to grow upwards; thus, measuring these pieces became impossible. Hence, further measurements were not obtained. Using the collected data, the mean growth rate was calculated during the first 48 h and the following 24 h according to the following formula, which was adapted for filamentous forms [28]:

*r* = (Ln*N*_*t*_ − Ln*N*_0_) × t^−1^, where *N*_t_ and *N*_0_ refer to the final and initial lengths of the mycelium, and *t* refers to time.

To determine whether there were significant differences in the mean growth rates of the fungus across the temperatures, we performed an ANOVA to analyze the first and the second measurements separately. The differences in the values of “*r*” at each temperature were then analyzed using an Unequal N HSD test. The numbers of cases were not equal because we did not include the wells in which the fungi were not visible or those in which the conidium degenerated. We also determined whether there were significant differences in the mean growth rates between the first and second measurements at each temperature using a *t* test.

To determine whether there were enough rotifers available for the fungus until the end of experiment, 96 h after the start of the experiment, the rotifers in each well were counted and categorized as active, inactive or caught by the fungus. Discriminating between active and inactive rotifers is important because according to our direct observations, in many cases, loricated rotifers can remain inactive for an extended time, but they are still alive and can regain movability.

On the same day, we also noticed that in certain wells, new conidia were produced by the fungus but only at a temperature of 20 °C. We checked the wells for fresh conidia again 3 days later.

We also wanted to determine the fate of the conidia that managed to trap a rotifer at the lowest temperature but did not grow during the experiment. Thus, the experimental wells were checked 11 and 30 days after the beginning of the experiment.

All observations were performed under an inverted Olympus microscope at a total magnification of ×200.

The statistical analysis was performed using STATISTICA software v 12.0 [29].

## Results

**Fig. 4:**
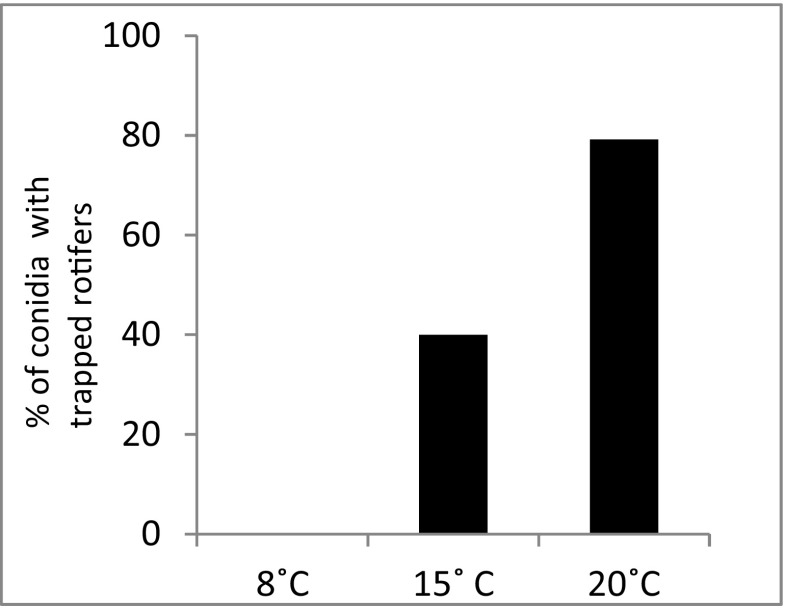
Percentage of conidia with trapped rotifers 24 h after the start of the experiment

The observations performed 24 h after presenting rotifers to the conidia revealed significant differences in the percentage of conidia that caught rotifers at each temperature. The conidia maintained at 8 °C did not catch any rotifers, whereas almost 80% of those maintained at 20 °C were successful. At 15 °C, the percentage of conidia that caught rotifers was intermediate (Fig. 4).

**Fig. 5:**
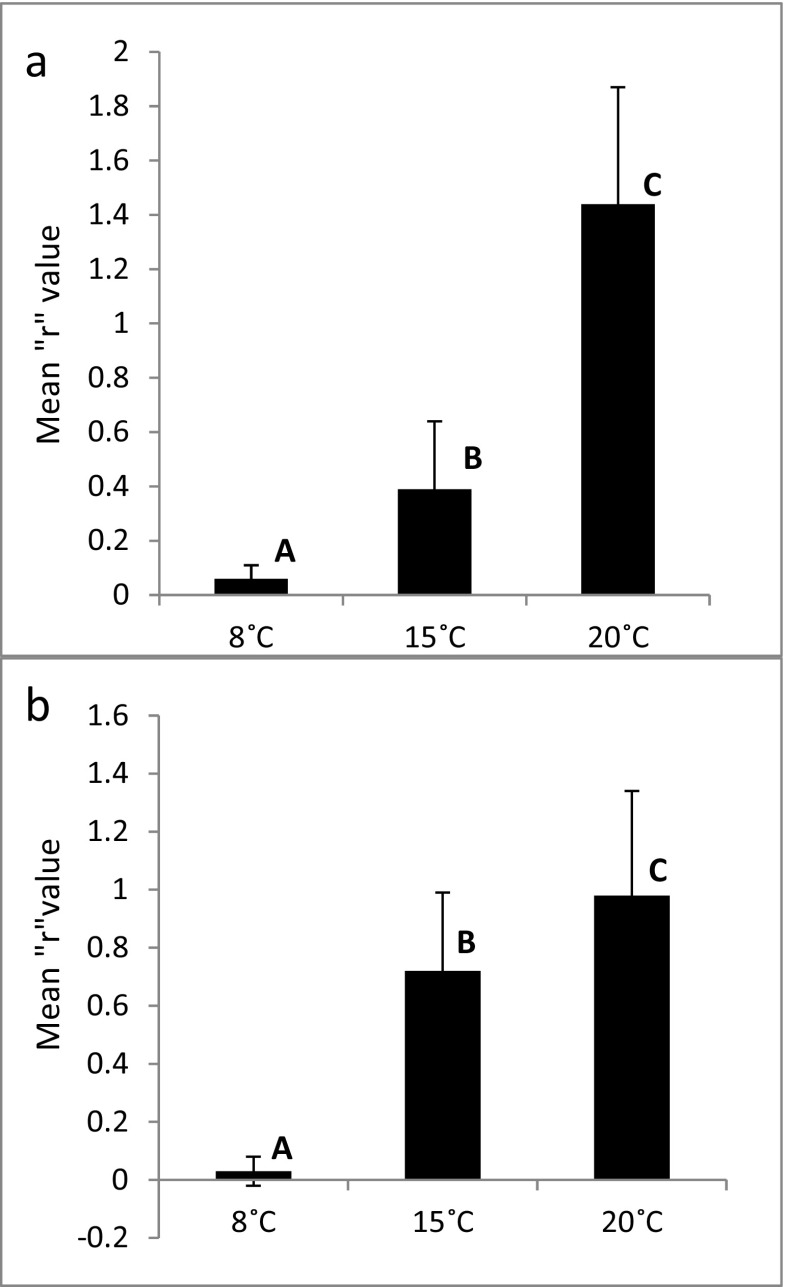
Mean “*r*” values after the first 48 h (**a**) and the following 24 h (**b**) at different temperatures. Capital letters indicate statistically significant differences. Bars indicate the standard deviations

The ANOVA showed that there were significant differences across the temperatures in the mean growth rate of the fungus after the first 48 h (*F*_2,47_ = 100.7, *p* < 0.001) and after the following 24 h (*F*_2,47_ = 52.49, *p* < 0.001). At 8 °C, the conidia did not grow during the experiment. The mean growth rate of the mycelium was nearly three times higher at 20 °C than that at 15 °C (Fig. 5). The Unequal N HSD test showed that the differences were significant at each of tested temperatures both after the first 48 h and the following 24 h (Fig. 5). Student’s *t* tests showed that the mean growth rate at 8 °C did not differ in time (*t*_14_ = 1.21, *p* = 0.25), whereas at higher temperatures, the differences across consecutive days were significant (*t*_15_ = 5.11, *p* < 0.001 at 15 °C and *t*_18_ = 2.77, *p* = 0.01 at 20 °C).

**Fig. 6:**
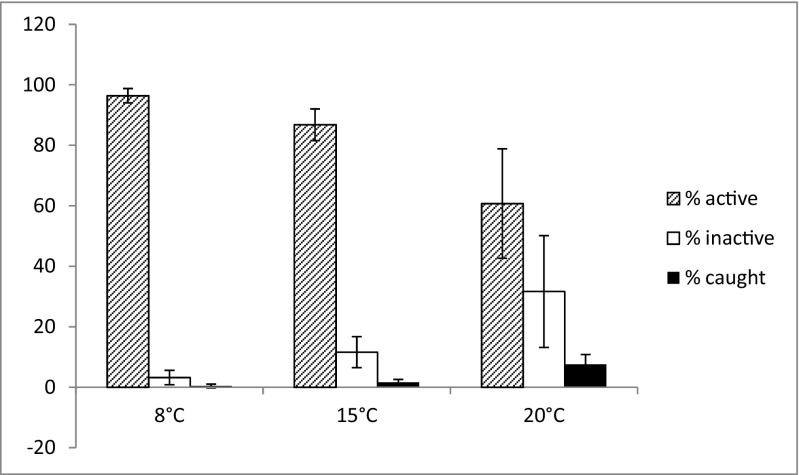
Mean fraction of active, inactive, and caught rotifers 96 h after the start of the experiment at each temperature. Bars indicate the standard deviations

Figure 6 shows the mean fractions of active, inactive, and caught rotifers calculated at the end of the experiment. The results show that at each temperature, the fraction of active rotifers was the highest. At the lowest temperature, the fraction of caught rotifers was extremely low. At 15 °C, the fractions of active, inactive, and caught rotifers were similar to those observed at 8 °C with slightly higher fractions of inactive and caught rotifers. At 20 °C, the fractions of inactive and caught rotifers were higher than those at both lower temperatures.

Direct observations performed on the fourth day of the experiment revealed that at a temperature of 20 °C, new conidia were produced in 73% of the wells, whereas at the two lower temperatures, no new conidia appeared. Three days later, new conidia were observed in 96% of the wells at 20 °C and 50% of the wells at 15 °C, but no freshly produced conidia were present at 8 °C.

The observations of the fungus at the lowest temperature showed that on the 11th day of the experiment, the conidia were alive in 21 of the 24 experimental wells, and the mycelium started to grow and reached a length in a range of 271 to 744 μm in 18 wells. In the three remaining wells, the conidia have not begun to elongate. On the 30th day of the experiment, the conidia that failed to elongate previously were deteriorated, whereas in the remaining wells, the growing mycelium reached a length in the range of 355 to approximately 4100 μm.

## Discussion

Predatory fungi are important, but until recently frequently overlooked, components of aquatic ecosystems. To date, not much is known regarding the influence of temperature on their life strategies even though temperature is considered an important factor influencing the significance of top-down and bottom-up effects in ecological communities [30, 31]. Our study is the first investigation of the effect of temperature on predatory fungus growth and conidia germination. Evidence indicates that the optimal temperature for the growth of nematode-trapping fungi is between 20 and 25 °C [9, 32], but no data are available regarding the influence of temperature on the development of conidia and the growth rate of species that belong to the genera *Lecophagus* and *Zoophagus* and prey on rotifers. These fungi belong to aquatic hyphomycetes and are characterized by the production of conidia (asexual spores). Conidia constitute an important stage of the life cycle for dispersion and survival in adverse environmental conditions [10, 33].

The germination of conidia in most filamentous fungi has been shown to be regulated by low molecular-mass nutrients, such as inorganic salts, sugars, and amino acids [33, 34]. However, not much is known regarding the conditions that determine conidia germination in predacious fungi, particularly those belonging to the genus *Lecophagus*. Tzean and Barron [11] showed that in *L. navicularis*, conidia develop short adhesive appendages in the presence of rotifer prey. Conidia of *L. vermicola* produce adhesive knobs that are capable of capturing nematodes [10]. Other nematophagous fungi produce so-called conidial traps when germinating in habitats where the level of nutrients is low and competition for nutrients among microorganisms is strong [3].

The results presented here indicate that the activity and development of conidia are strictly temperature-dependent. Apparently, a temperature of 20 °C is the most favorable. At this temperature, most conidia managed to trap a rotifer during the first 24 h of the experiment, whereas at the lowest temperature, no conidium was successful (Fig. 4). Although it has been reported that in the presence of rotifers, conidia of certain *Lecophagus* sp. germinate to produce adhesive pegs for trapping rotifers [35] and rotifers and tardigrades are attracted to adhesive pegs [13], no extensive studies investigating the relationship between conidia and their prey have been conducted. Our results clearly indicate that a temperature of 8 °C significantly slows the process of conidia germination. It might be argued that the rotifers may be less active at this temperature, but as even at the end of the experiment, the percentage of active rotifers at 8 °C remained high (Fig. 6), their reduced motility cannot be the only reason preventing the conidia from trapping the prey. Furthermore, if the conidia actually attract rotifers, their relatively limited motility should not prevent their capture, particularly when there were approximately 100 rotifers available for each conidium (Fig. 6). Apparently, higher temperatures enhance the trap formation and ability to trap the rotifers. It appears that because at 8 °C, the conidia were barely able to catch rotifers and many conidia deteriorate over time, this temperature could limit the development and dispersion of this species in the environment. However, while the conidia did not germinate and trap any prey during the first 24 h at the lowest temperature, on the 30th day, the percentage of conidia that died was below 50%. The remaining conidia managed to trap rotifers and eventually began to develop into quite long mycelium. This result shows that the fungus can grow at temperatures as low as 8 °C. Because some rotifers can live and even proliferate at such a low temperature [25], the fungus can be expected to survive in adverse conditions during the winter season.

The comparison of the mycelium growth rate at different temperatures shows that the higher the temperature, the higher the growth rate, whereas the lowest temperature considerably limits the growth of *Lecophagus* (Fig. 5). The growth rate at the highest and medium temperatures changed across the consecutive days of the experiment. At 20 °C, the fungus growth was faster during the first day and slowed on the following day. The opposite tendency was observed at the medium temperature. At the lowest temperature, the mycelium began to grow, but the process was very slow and practically did not change across the days. The temperature of 8 °C was also shown to be a limiting factor for the growth of the *L. inermis* rotifers. Apparently, the temperature-dependent development of predatory fungi is ecologically coupled to the life-history of its prey. The decreasing growth rate at 20 °C might have been caused by the decreasing percentage of active rotifers. Because the fungus grows quickly and its growth is strongly dependent on the availability of prey organisms, the fungus can quickly overexploit its resources. At medium temperatures, the whole process is slower, allowing the high growth rate to persist. Another factor might have also influenced the growth rate of the fungus at the highest temperature. On the day we quantified the percentage of active rotifers, we also observed that in 16 of the 24 wells at 20 °C, the hyphae began to produce new conidia, whereas no new conidia were observed at 15 or 8 °C. The production of spores in aquatic hyphomycetes was shown to depend on the temperature and nutrients, among other factors. The increase in the temperature and nutrient concentrations results in intensified growth and sporulation [1, 36]. Again, the data did not include predatory fungi with possibly different physiological reactions. In our experiment, no additional nutrients were added, but because predatory fungi depend mainly on trapped prey, increases in the number of trapped rotifers should result in a higher conidia production. Thus, the influence of temperature on conidia production might be indirect.

In the case of aquatic hyphomycetes, fungi have been shown to allocate up to 80% of their production to conidia [37]. If similar processes occur in predatory fungi, these processes could explain why the growth of the mycelium at the highest temperature rapidly slowed. However, Smith [38] suggested that the growth of the mycelium and sporulation in filamentous fungi are cellular processes that compete for limited metabolic factors rather than being mutually exclusive phenomena. If vegetative growth and sporulation do not occur simultaneously, the limited vegetative growth may be caused by nutrient shortages [38]. Therefore, our results suggest that at the highest temperature, the fungus used most of the nutrients during the first 48 h.

Our experiment illustrates the possible predator-prey dynamics at average temperature ranges in a moderate climate. Apparently, the most stable coexistence of predatory fungus and its rotifer prey is possible at a temperature of approximately 15 °C, whereas at a temperature as low as 8 °C, the fungus development is limited mainly by its temperature-dependent internal growth rate. At the highest temperature of 20 °C, the rotifer population is strongly limited by the predatory fungus, and then, the fungus growth is limited by the shortage of active rotifers. This hypothesis is consistent with conclusions drawn by Hoekman [30], who stated that a higher temperature may increase the strength of top-down effects. Therefore, the understanding of how communities respond to changes in temperature is a challenge in community ecology. These phenomena are particularly pronounced in the biocenoses of wastewater treatment plants. The biological processes present in WWTPs provide perfect conditions for the fast co-evolution of activated sludge inhabitants due to nearly unlimited nutrient resources. Therefore, studies investigating the communities evolving in these systems could help predict how the predator-prey dynamics evolve in changing environments.

## Electronic supplementary material

Vid. 1 Video sequence depicting the moment of conidia dispersion (AVI 33.5 mb)
